# Metachronous immune-related adverse events involving type 1 diabetes and isolated adrenocorticotropic hormone deficiency associated with pembrolizumab monotherapy for metastatic head and neck cancer: a case report

**DOI:** 10.1186/s13256-023-04106-6

**Published:** 2023-09-12

**Authors:** Hiroaki Iijima, Akihiro Sakai, Koji Ebisumoto, Mayu Yamauchi, Takanobu Teramura, Aritomo Yamazaki, Toshihide Inagi, Kenji Okami

**Affiliations:** Department of Otolaryngology, Head and Neck Surgery, Tokai University, Isehara, Japan

**Keywords:** Immune checkpoint inhibitors, Head and neck cancer, Recurrence/metastasis, Diabetic ketoacidosis, Pituitary

## Abstract

**Background:**

Immune checkpoint inhibitors might cause immune-related adverse events that are still largely unknown.

**Case presentation:**

An 80-year-old Asian female was diagnosed with cervical lymph node metastasis from lip cancer (cT1N0M0) and underwent right cervical neck dissection. Subsequently, she developed right cervical lymph node relapse and lung metastasis. The patient was deemed eligible for pembrolizumab owing to unresectable neck recurrence and pulmonary metastasis. The Combined Positive Score of the submandibular lymph nodes was 100. Pembrolizumab monotherapy was initiated, and complete remission was achieved. She developed diabetic ketoacidosis in the eighth month and was diagnosed with fulminant type 1 diabetes mellitus. Insulin induction was performed. The patient developed adrenal insufficiency after 10 months. These were immune-related adverse events, caused by pembrolizumab. The patient has remained in complete remission, and pembrolizumab therapy was continued.

**Conclusions:**

The study presents the first reported case of type 1 diabetes in a patient with head and neck squamous cell carcinoma treated with pembrolizumab monotherapy, in Japan. Efficient interdepartmental collaboration will promote the management of severe immune-related adverse events and improve the quality of life of patients.

## Background

Immune checkpoint inhibitors (ICIs) such as nivolumab and pembrolizumab were approved in Japan in March 2017 and December 2019, respectively, for the treatment of recurrent or metastatic head and neck squamous cell carcinoma (HNSCC). ICIs might cause immune-related adverse events (irAEs), which are significantly different from those of conventional chemotherapy; however, the irAEs are still largely unknown. Although there have been several reports of type 1 diabetes and isolated adrenocorticotropic hormone (ACTH) deficiency in various cancers [1], the incidence of these irAEs in head and neck cancers is very rare. In this report, we present a case of metachronous development of irAEs with type 1 diabetes and ACTH deficiency associated with monotherapy for metastatic HNSCC. The study presents the first reported case of type 1 diabetes in a patient with head and neck squamous cell carcinoma (HNSCC) treated with pembrolizumab monotherapy, in Japan.

## Case presentation

An 80-year-old Japanese Asian female with postoperative relapse of lip cancer (T1N0M0) presented with a cervical mass, following a referral by the department of plastic surgery. She had medical history of small intestine carcinoid at 67 years of age.

**Fig. 1:**
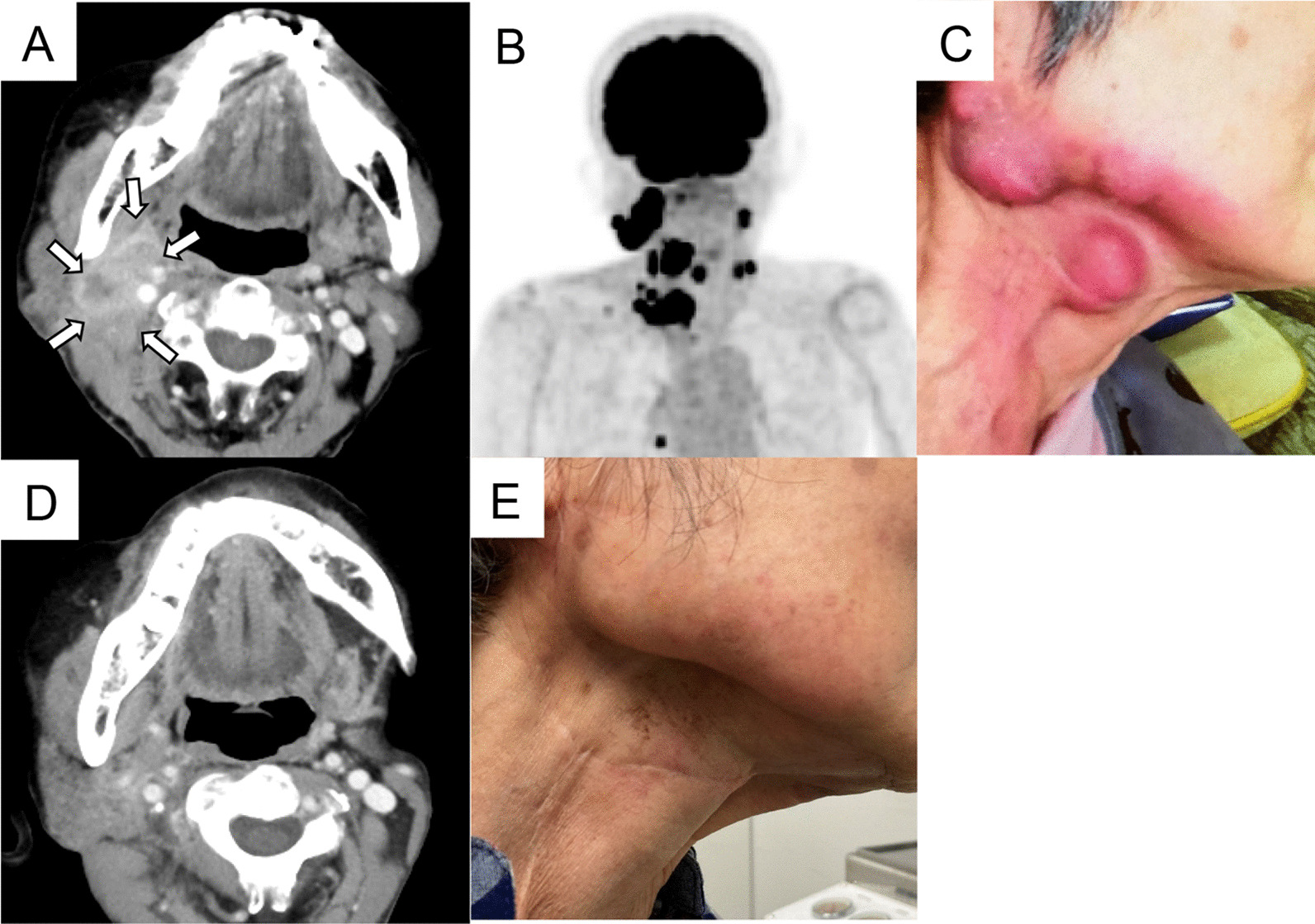
Computed tomography (CT) and positron emission tomography-computed tomography (PET-CT) course. Two months after right cervical dissection for right submandibular lymph node metastasis, computed tomography revealed right cervical recurrence and lung metastasis (**A**), positron emission tomography-computed tomography demonstrated multiple cervical metastases (**B**), and skin erythema associated with tumor invasion (**C**). Five months after initiation of pembrolizumab therapy, computed tomography revealed marked shrinkage of the tumor (**D**), which was evaluated as complete remission (CR), and skin erythema was absent (**E**)

Ten years before the first presentation, she underwent treatment for lip cancer (T1N0M0) and skin tumor excision with local skin flap reconstruction at the department of plastic surgery. The pathological diagnosis was squamous cell carcinoma. After being recurrence-free for 9 years, she was referred to our department owing to lymph node swelling in the right submandibular region. She was diagnosed with cervical lymph node metastasis from lip cancer and underwent right cervical neck dissection. Two months after surgery, the patient developed right cervical lymph node relapse and lung metastasis (Fig. 1A–C). We prescribed ICI for unresectable cervical recurrence and lung metastases. The combined positive score (CPS) of the resected submandibular lymph nodes was 100, and pembrolizumab monotherapy was initiated. The administration dose was 200 mg/body weight at 3-week intervals.

With the initiation of pembrolizumab administration, the cervical lymph node swelling started subsiding, and 5 months after ICI treatment, the tumor was absent on computed tomography (CT) (Fig. 1D) and the skin findings improved (Fig. 1E), showing complete remission (CR), which was maintained with the continuation of pembrolizumab monotherapy.

**Fig. 2:**
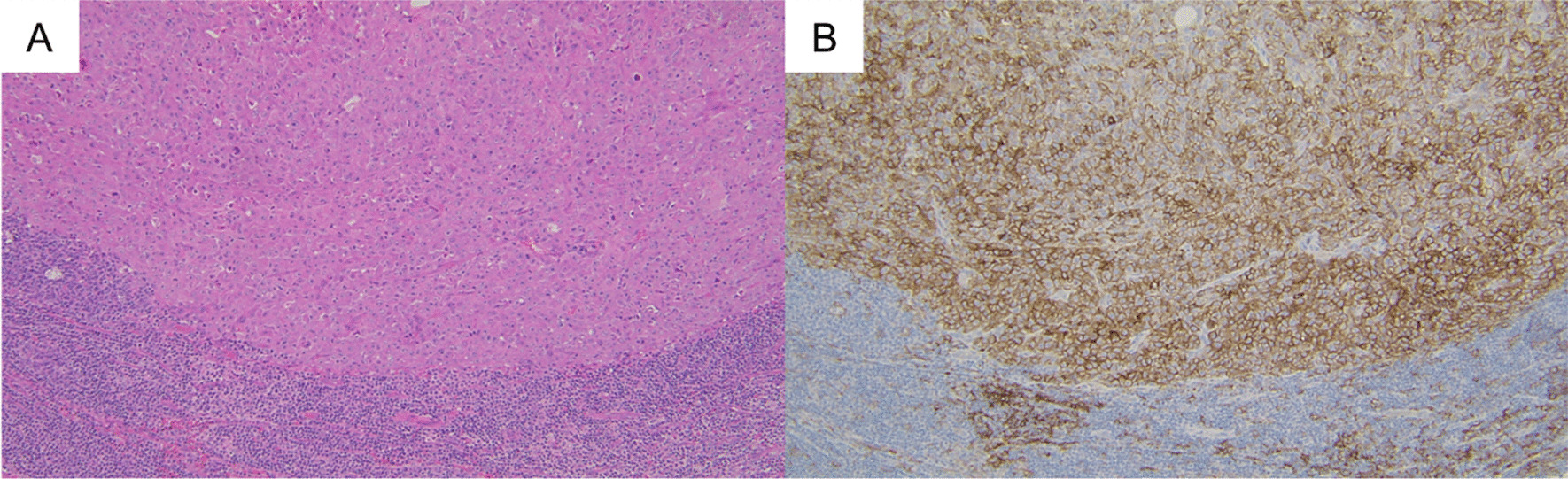
Histopathology of the submandibular lymph nodes. Hematoxylin and eosin (HE) staining **A** and immunostaining with PD-L1 IHC 22C3 pharmDx assay (Agilent Technologies, Santa Clara, CA, USA) (**B**). The combined positive scoring (CPS) count was 100

Histopathology of the submandibular lymph nodes is presented in Fig. 2.

On the 13th course of pembrolizumab administration (day 252), the patient complained of ill health with common cold symptoms and vomiting for 2–3 days. Blood tests (Table 1) revealed a blood glucose level of 407 mg/dl, which was previously unrecognized as a high blood glucose level. Although the HbA1c level was 6.6%, urine analysis revealed urinary glucose as 4+ and urinary ketones as 3+ . Laboratory tests 3 weeks earlier showed no evidence of diabetes. The sudden onset of diabetes mellitus was thought to be fulminant type 1 diabetes. We immediately consulted the department of nephrology, metabolism, and endocrinology for diabetic ketoacidosis (DKA) and fulminant type 1 diabetes mellitus. She was diagnosed with fulminant type 1 diabetes mellitus due to irAEs. She was treated under emergency hospitalization. Her general condition and level of consciousness were stable and was treated with continuous intravenous insulin and external fluid administration. After approximately 1 month of inpatient care, the patient was discharged from the hospital, pembrolizumab monotherapy was resumed with insulin treatment, and CR was maintained.

**Table 1:** Blood parameters tested on day 252

Blood cell count		Biochemistry		Urine analysis		Venous blood gas	
WBC	11,100/μℓ	Alb	4.0 g/dl	Protein	±	pH	7.237
RBC	344 104/μℓ	CK	75I U/L	Glucose	4 +	PCO2	30.7
Hb	11.8 g/dℓ	AST	18 IU/L	Specific gravity	1.031	PO2	101.0
Ht	36.3%	ALT	15 IU/L	pH	5.5	HCO3-	12.6
MCV	105.5 fl	LDH	204 IU/L	Urobilinogen	normal	Anion Gap	28.4
PLT	23.0 104/μℓ	ALP	58 IU/L	Bilirubin	(−)		
		Cr	0.60 mg/dl	Ketone body	3 +		
		BUN	28 mg/dl	Leukocytes	(−)		
		Glu	407 mg/dl	Nitrous acid	(−)		
		Na	136 mEq/L	Occult blood	(−)		
		K	5.2 mEq/L				
		Cl	97 mEq/L				
		CRP	5.337 mg/dl				
		HbA1c	6.6%				

*WBC* white blood cells, *RBC* red blood cells, *Hb* hemoglobin, *Ht* hematocrit, *MCV* mean corpuscular volume, *PLT* platelets, *Alb* albumin, *CK* creatine kinase, *AST* aspartate transferase, *ALT* alanine transaminase, *LDH* lactate dehydrogenase, *ALP* alkaline phosphatase, *Cr* creatinine, *BUN* blood urea nitrogen, *CRP* c-reactive protein

During the visit for the 15th course of pembrolizumab (day 294), the patient complained of nausea and general fatigue for 1 week. The blood pressure was low (96/49 mmHg); however, the other vital signs were stable. Suspecting adrenal insufficiency, an urgent blood test was performed, which revealed hyponatremia (130 mmol/l), hypoglycemia (57 mg/dl), low cortisol (1.18 µg/dl), and normal ACTH 7.5 pg/ml (Table 2). She was immediately referred to the department of nephrology, metabolism, and endocrinology and was admitted immediately. No pituitary enlargement was noted on magnetic resonance imaging (MRI) of the head. A triad load test [thyrotropin-releasing hormone (TRH), luteinizing hormone-releasing hormone (LHRH), and corticotropin-releasing hormone (CRH)] was performed, and ACTH levels were 1.05 pg/ml before loading, 5.42 pg/ml at 30 min, and 7.67 pg/ml at 60 min, which led to the diagnosis of isolated ACTH deficiency. She was administered 20 mg of hydrocortisone and was discharged when her general condition stabilized.

**Table 2:** Blood parameters tested on day 294

Blood cell count		Biochemistry		Endocrine	
WBC	5400/μℓ	Alb	3.2 g/dl	ACTH	7.5 pg/ml
Seg	37.3%	CK	43 IU/L	Cortisol	1.18 μg/dl
Stab	Seg + stab%	AST	29 IU/L	TSH	7.7 IU/L
Hb	9.6 g/dℓ	ALT	14 IU/L	FreeT3	3.83 IU/L
PLT	18.110^4^/μℓ	LDH	196 IU/L	FreeT4	0.99 IU/L
		ALP	83 IU/L		
		Cr	0.45 mg/dl		
		BUN	8 mg/dl		
		Glu	57 mg/dl		
		Na	130 mEq/L		
		K	3.5 mEq/L		
		Cl	95 mEq/L		
		CRP	< 0.09 mg/dl		

*WBC* white blood cells, *seg* segmented neutrophils, *Hb* hemoglobin, *Ht* hematocrit, *MCV* mean corpuscular volume, *PLT* platelets, *Alb* albumin, *CK* creatine kinase, *AST* aspartate transferase, *ALT* alanine transaminase, *LDH* lactate dehydrogenase, *ALP* alkaline phosphatase, *Cr* creatinine, *BUN* blood urea nitrogen, *CRP* c-reactive protein, *ACTH* adrenocorticotropic hormone, *TSH* thyroid stimulating hormone

Pembrolizumab monotherapy was continued, and CR was maintained for 540 days after ICI treatment was started.

## Discussion and conclusions

IrAEs occur in 70–80% of patients treated with ICIs and occur within 3–6 months after initiation of treatment [2]. In addition to interstitial lung disease, pituitary dysfunction, and skin disorders, conditions such as thyroid dysfunction, type 1 diabetes mellitus, and myasthenia gravis are involved.

The frequency of endocrine-related irAEs induced by ICI for various types of carcinomas is summarized in Table 3 [3, 4]. Hypothyroidism is the most frequent endocrine-related irAE, adrenal insufficiency observed in the present case ranges from 0–4.5%, and type 1 diabetes is very rare.

**Table 3:** Summary of the endocrine-related immune-related adverse events (irAE) frequency associated with immune checkpoint inhibitors (ICI) in various carcinomas (modified from refs. 3, 4)

Drug name		Frequency of endocrine-related irAEs (%)			
		Pituitary disorder	Hypothyroidism	Adrenal insufficiency	Type 1 diabetes
Anti PD-L1	Nivolumab	0 ~ 0.9	10	0 ~ 3.3	0.25
	Pembrolizumab	0 ~ 1.2	10 ~ 20	0 ~ 4.3	Case reports exist

Multiorgan irAEs have been reported previously [5], which include lung disorders, acute hepatitis, skin disorders, and colitis. In the present case, type 1 diabetes mellitus and isolated ACTH deficiency (adrenal insufficiency) developed metachronously. To the best of our knowledge, there have been no reports of these two irAEs occurring metachronously in a single patient. This occurrence is considered extremely rare.

Adrenal insufficiency (isolated ACTH deficiency) with pembrolizumab was observed in one case (on day 163) when pembrolizumab monotherapy was administered in the KEYNOTE048 study [6] for head and neck cancer; however, there was no such case reported in the chemotherapy combination group. In pituitary disorders caused by anti-PD-L1 antibodies, there was no swelling of the pituitary gland associated with inflammation and only reduced ACTH secretion was reported [7]. In contrast, anti-CTLA-4 antibodies such as ipilimumab lead to panhypopituitarism, with MRI showing pituitary enlargement and an onset at approximately 10 weeks [8]. The occurrence of pituitary inflammation (hypophysitis) is relatively rare during treatment with anti-PD-1 antibodies (0–0.9%) and anti-CTLA-4 antibodies such as ipilimumab (0–10%). The reasons for this difference in frequency and clinical features remain unclear [9].

There were no cases of type 1 diabetes mellitus associated with pembrolizumab reported in the KEYNOTE048 study [5], either as monotherapy or in combination with chemotherapy. There was only one case of HNSCC reported in the KEYNOTE012 study in a different patient population [10]. In the KEYNOTE 181 study of esophageal cancers, the onset time was similar to that in the present case (252 days) [7]. Type 1 diabetes mellitus is reported to be less frequent but a more severe form of irAE [11]. The incidence of type 1 diabetes in Japan with nivolumab, a similar PD-L1 inhibitor as pembrolizumab, was reported to be 0.33%, with a mean median age of 63 years and a mean time from nivolumab treatment to the onset of type 1 diabetes of 155 days [12]. Deaths from fulminant type 1 diabetes and diabetic ketoacidosis associated with pembrolizumab have also been reported [13]. It is essential to monitor blood glucose, perform urinalysis frequently, and detect atypical but suggestive findings of type 1 diabetes and ketoacidosis, such as common cold symptoms, gastrointestinal symptoms, and general fatigue, as in the present case. Moreover, educating patients about the symptoms of hyperglycemia and diabetic ketoacidosis, such as dry mouth, polyuria, general fatigue, nausea, vomiting, and abdominal pain, is necessary. HbA1c levels may not be high in patients with fulminant type 1 diabetes. An immediate consultation system for various irAE target organs and early intervention with an interdisciplinary approach must be considered.

The KEYNOTE048 study demonstrated that, the higher the CPS, as in the present case, the better the anti-tumor effect of pembrolizumab [6, 14]. The association between the irAEs of pembrolizumab and CPS in our department is presented in Table 4. IrAEs occurred more frequently in patients with CPS > 20, with a frequency of 12% (5 events, 4/33 cases; adrenal insufficiency, 2 cases; hypothyroidism, 1 case; drug-induced hepatitis, 1 case; and type 1 diabetes, 1 case). Some reports have revealed that irAEs are associated with the antitumor effects of drugs [15]. The CPS, in this case, was as high as 100, which may have facilitated the antitumor effect and led to irAEs; however, further investigation is required in this aspect.

**Table 4:** Association between immune-related adverse events (irAEs) by pembrolizumab and combined positive scoring (CPS) in our department

	CPS < 1	1 ≤ CPS < 20	20 ≤ CPS
Number of irAE cases/number of patients administered pembrolizumab	0/5	0/8	4/20
Incidence of irAEs	0%	0%	20%

This is the first report of metachronous IrAEs involving type 1 diabetes and isolated ACTH deficiency, associated with pembrolizumab monotherapy for metastatic head and neck cancer. IrAEs with pembrolizumab occur in various patients but are not rare. It is necessary to be familiar with each irAE and be aware of its characteristics. Keen observation of the symptoms and parameters associated with irAEs is necessary. Immediate and appropriate collaboration with other specialists is considered significant for the management of severe irAEs to improve the quality of life of patients.

## Data Availability

The data supporting the findings of this case report are available from the corresponding author upon reasonable request.

## Abbreviations

ICIs: Immune checkpoint inhibitors
irAEs: Immune-related adverse events
CR: Complete remission
HNSCC: Head and neck squamous cell carcinoma
ACTH: Adrenocorticotropic hormone
CPS: Combined positive score
CT: Computed tomography
MRI: Magnetic resonance imaging
TRH: Thyrotropin-releasing hormone
LHRH: Luteinizing hormone-releasing hormone
CRH: Corticotropin-releasing hormone
